# A second HD mating type sublocus of *Flammulina velutipes* is at least di-allelic and active: new primers for identification of HD-a and HD-b subloci

**DOI:** 10.7717/peerj.6292

**Published:** 2019-02-22

**Authors:** Wei Wang, Irum Mukhtar, Tiansheng Chou, Siyuan Jiang, Xinrui Liu, Arend F. van Peer, Baogui Xie

**Affiliations:** 1 Mycological Research Center, College of Life Sciences, Fujian Agriculture and Forestry University, Fuzhou, Fujian, China; 2 Shandong Provincial Key Laboratory of Agricultural Microbiology, College of Plant Protection, Shandong Agricultural University, Tai’an, China; 3 Mushroom Research Group, Plant Breeding, Wageningen University and Research, Wageningen, The Netherlands

**Keywords:** *Flammulina velutipes*, Homeodomain, Primers, Di-allelic, HD subloci, Mating pathway

## Abstract

**Background:**

Sexual development in *Flammulina velutipes* is controlled by two different mating type loci (HD and PR). The HD locus contains homeodomain (Hd) genes on two separate HD subloci: HD-a and HD-b. While the functionality of the HD-b sublocus has been largely confirmed, the status and content of the HD-a sublocus has remained unclear.

**Methods:**

To examine the function of the HD-a sublocus, genome sequences of a series of *F. velutipes* strains were analyzed and tested through series of amplification by specific primer sets. Furthermore, activity of di-allelic HD-a locus was confirmed by crossing strains with different combinations of HD-a and HD-b subloci.

**Results:**

Sublocus HD-b contained a large variety of fixed Hd1/Hd2 gene pairs, while the HD-a sublocus either contained a conserved Hd2 gene or, a newly discovered Hd1 gene that was also conserved. Identification of whole HD loci, that is, the contents of HD-a and HD-b subloci in a strain, revealed that strains with similar HD-b subloci could still form normal dikaryons if the two genes at the HD-a sublocus differed. At least di-allelic HD-a sublocus, is thus indicated to be actively involved in mating type compatibility.

**Conclusions:**

HD-a sublocus is active and di-allelic. Using the new information on the HD subloci, primers sets were developed that specifically amplify HD-a or HD-b subloci in the majority of *F. velutipes* strains. In this way, unknown HD mating types of *F. velutipes* can now be quickly identified, and HD mating type compatibility conferred by HD-a or HD-b can be confirmed by PCR.

## Introduction

Mating in basidiomycetous fungi is controlled by sets of specialized genes that are located at mating type (MAT) loci called MAT-A and MAT-B (Raper, 1966; Brown & Casselton, 2001; Raudaskoski & Kothe, 2010; Kües, James & Heitman, 2011; Raudaskoski, 2015), more recently referred to as HD (homeodomain) and PR (pheromone receptor) loci, respectively (Maia et al., 2015; Wang et al., 2016). Genes at these loci control the HD or PR mating type pathways that regulate various steps in the formation of a dikaryon and in sexual reproduction (Kües, James & Heitman, 2011; Kües, 2015; Maia et al., 2015; Coelho et al., 2017). As such, these loci and their corresponding genes are of importance for the general understanding of the sexual life cycle of basidiomycetes, and for practical applications regarding mushroom breeding and production.

Homeodomain and PR loci can contain several sets of mating type genes that are located on subloci. These subloci are commonly di-or multiallelic when active (Kües, James & Heitman, 2011; Kües, 2015; Coelho et al., 2017). Heterozygosity at one of the subloci within the HD and one of the subloci in the PR locus, is generally sufficient to activate the corresponding mating type pathways, and does so in *Flammulina velutipes* (Kües, 2015; Wang et al., 2016). The genetic structure and control of the PR mating type pathway of *F. velutipes* has been attributed to two PR subloci: PR-a and PR-b. The PR-a and PR-b-subloci are located on chromosome 3 at a distance of about 180 kb (van Peer et al., 2011; Wang et al., 2016). Both PR subloci have been found to be multiallelic, and to contain varying combinations of *pheromone receptor* (*FvSTE3*) and *pheromone precursor* (*FvPp*) genes. Each of the PR subloci has the potential to activate the PR pathway (Wang et al., 2016).

Understanding of the HD locus in *F. velutipes* has remained incomplete. The HD locus of *F. velutipes* consists of two apparent subloci: HD-a and HD-b. These subloci are located on chromosome 8 at a distance of 73 kb, and generally segregate as a whole (van Peer et al., 2011; Wang et al., 2016). The separation of the two subloci has been traced to two large inversion within the overall highly syntenic HD region (van Peer et al., 2011; James et al., 2004). While the archetypal association of the *mitochondrial intermediate peptidase* (*MIP*) gene with the HD locus has been maintained, the *Beta-flanking* (*Bfg*) *gene* has been separated. This latter, together with the large distance between the HD-a and HD-b subloci, has complicated the design of conserved primers to easily obtain and identify *F. velutipes* HD loci (James et al., 2004; Kües, James & Heitman, 2011; van Peer et al., 2011). The genes for the two classes of HD proteins at the HD-b sublocus, *FvHd1* and *FvHd2*, display a common organization with oppositely, outward oriented transcription directions (James et al., 2004; Kües, James & Heitman, 2011; van Peer et al., 2011; Wang et al., 2016). Conservation of the C-terminal regions of *F. velutipes* HD1 and HD2 proteins the HD-b sublocus was found to be particularly high in comparison with other species, yet heterozygosity at the HD-b sublocus clearly conferred activation of the HD mating type pathway (Wang et al., 2016).

The HD-a sublocus of *F. velutipes* was initially found to contain a single *FvHd2* gene (*FvHd-a2-1*) that was conserved amongst tested *F. velutipes* strains (L11, W23, and KACC42780) in which an HD sublocus was detected in addition to HD-b. The encoded FvHD_A_2-1 protein was clearly distinct from the HD2 proteins of the HD-b sublocus, and did not confer cross activation with HD1 proteins of the HD-b sublocus. While different from HD-b, functionality of the HD-a sublocus remained unclear. Moreover, research revealed that *F. velutipes* strains existed without the conserved *FvHd-a2-1* gene (Wang et al., 2016), suggesting several possibilities: (1) absence of the entire HD-a sublocus, (2) the existence of another HD sublocus, or (3) different genes at the HD-a sublocus, making the sublocus multiallelic and possibly functional. In this study, we determined the unknown contents and functionality of HD subloci in *F. velutipes* strains that did not have the *FvHd-a2-1* gene. We present a detailed structure of the HD locus in *F. velutipes*, and report primer sets with which the contents of both HD subloci in *F. velutipes* can be amplified.

## Material and Methods

### Maintenance and sequencing of *F. velutipes* strains

A total of 17 monokaryotic *F. velutipes* strains (Fv25-1, Fv-24, Fv25-3, Fv25-4, Fv27-3, Fv-27-1, Fv12-2, NJ6-21, NJ6-3, Fv10-4, Fv01-10, Fv10(Col)-4, Fv34-1, Fv34-18, L22, L11, and W23) were obtained from the Fujian Edible Fungi Germplasm Resource Collection Center of China and were maintained on 2% potato dextrose agar medium (PDA; 200 g/L potato; 20 g/L glucose; 20 g/L agar) at 25 °C. Genomic DNA (gDNA) was extracted from strains by using a 2% CTAB method (van Peer et al., 2011). Mycelium of each strain was grown separately on cellophane membranes and was used for gDNA extraction followed by sequencing. gDNA of seven strains (Fv25-1, Fv25-3, NJ6-21, NJ6-3, Fv34-1, Fv34-18, and L22) was sent for sequencing. Paired-end (90 bp) DNA libraries with 500 bp inserts were generated and sequenced (Illumina GAII platform, Zhejiang California International Nano Systems Institute, Hangzhou, China). Clean reads (six GB per genome, corresponding to ∼170 times coverage) were assembled into separate draft genomes by using SPAdes3.10.1 Linux with default parameters. Assembled draft genomes were converted for use in standalone blast using blast freeware (ncbi-blast-2.2.31, https://blast.ncbi.nlm.nih.gov/blast.cgi). Genome sequences of L11 and W23 strains were obtained from previous studies (Zeng et al., 2015; Wang et al., 2015).

### Identification of *Hd* mating type genes and phylogenetic analysis of predicted proteins

Draft genome sequences of *F. velutipes* strains (Fv25-1, Fv25-3, NJ6-21, NJ6-3, Fv34-1, Fv34-18, and L22) were screened for *Hd* mating type genes with tBLASTn by using known *F. velutipes* HD proteins as query. Accession numbers of previously reported HD genes (van Peer et al., 2011; Wang et al., 2016) are mentioned in Table 1. Contigs/scaffolds of draft genomes that matched with HD proteins or *MIP* genes, were selected. *Hd* genes were annotated by following gene models of previously identified *Hd* genes and Hd proteins (van Peer et al., 2011; Wang et al., 2016). *Fv_Hd_A_1-1* was predicted with the help of ZOOMlite software, and annotated in genomes of *F. velutipes* strains. Genes related to HD-a and HD-b subloci are mentioned in Table 2.

**Table 1:** GenBank accession numbers of the *Hd* and *MIP* genes of *F. velutipes* (van Peer et al., 2011; Wang et al., 2016).

Gene	GeneBank accession number	Gene	GeneBank accession number
*Hd1-1/Hd2-2^KACC42780^*	HQ630588.1	*Hd1-3/Hd2-4^L11^*	KC208594.1
*Hd2-1/MIP^KACC42780^*	HQ630589.1	*Hd2-1/MIP^L11^*	KC208595.2
*Hd1-2/Hd2-3^W23^*	KC208604.1	*Hd1-4/Hd2-5^27-1^*	KT808673.1
*Hd2-1/MIP^W23^*	KC208603.2	*Hd1-5/Hd2-6^25-1^*	KT808672.1
*Hd1-6^27-3^*	KT808676.1	*Hd2-8^20-2^*	KT808678.1
*Hd2-7^27-3^*	KT808677.1	*Hd2-9^25-4^*	KT808679.1
*FvHd1-7^25-3^*	KU852595.1	*–*	–

**Note:**

Strain names are indicated with superscript.

**Table 2:** List of assembled contigs that contained putative HD-a and HD-b subloci, in draft genomes of *F. velutipes* strains generated during this study.

Strain	Node # in genome	Length (bps)	HD sublocus
Fv25-1	Node-30	247,924	HD-b (*FvHd1-5,FvHd2-*6)
	Node-50	196,420	HD-a (*FvHd2-1*) & *MIP*
Fv25-3	Node-9	312,936	HD-a (*FvHd1-1*) & *MIP*; HD-b (*FvHd1-3 FvHd2-4*)
Fv34-1	Node-1	848,524	HD-a (*FvHd1-1*) & *MIP*; HD-b (*FvHd1-4 FvHd2-5*)
Fv34-18	Node-12	265,899	HD-b (*FvHd1-2, FvHd2-3*)
	Node-18	225,972	HD-a (*FvHd2-1*) & *MIP*
NJ6-21	Node-1	1,074,319	HD-a (*FvHd1-1*) & *MIP*: HD b (*FvHd1-4,FvHd2-5*)
NJ6-3	Node-111	10,267	HD-a (*FvHd2-1*) & *MIP*
	Node-25	182,944	HD-b (*FvHd1-4, FvHd2-5*)
L22	Node-3	502,672	HD-a (*FvHd2-1*) & *MIP;* HD-b (*FvHd1-2 FvHd2-3*)

Nuclear localization signals (NLS), dimerization motifs (Di), and HD in HD mating type proteins were predicted by PSORT II Prediction (http://psort.hgc.jp/form2.html; Horton et al., 2007), COILS Server (http://embnet.vital-it.ch/software/COILS_form.html; Windows width = 14; Lupas, Van Dyke & Stock, 1991) and MOTIF Search (http://www.genome.jp/tools/motif/). Protein sequences of *Hd* genes were aligned using MUSCLE (Edgar, 2004). Phylogenetic analysis was performed with MEGA 6.0 by using default program settings and 1,000 Bootstrap value to construct a neighbor-joining tree (Tamura et al., 2013).

### Crossing of strains with different HD subloci

**Figure 1:**
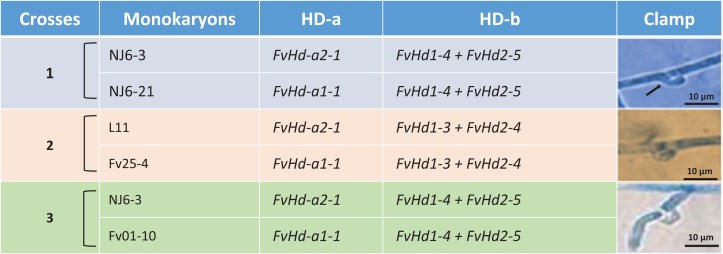
Detail of three different crosses between five *F. velutipes* strains (NJ6-3, NJ6-21, L11, Fv25-4, Fv01-10) with different HD-a but similar HD-b subloci. Arrowhead (black) indicates clamp connection in dikaryotic hypha.

**Figure 2:**
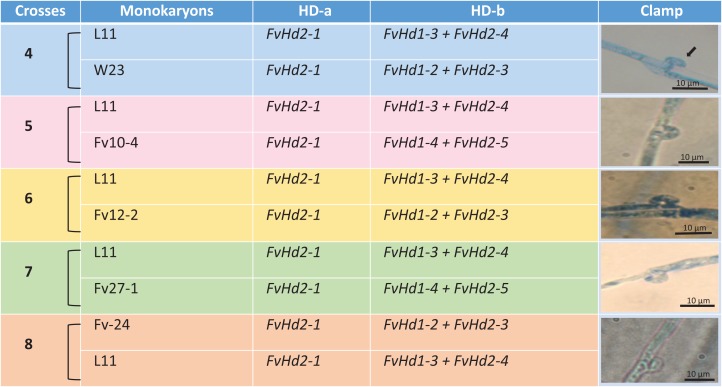
Crosses (4–8) between six *F. velutipes* strains (L11, W23, FV12-2, FV27-, Fv01-4, Fv-24) with similar HD-a but different HD-b subloci. Arrowhead (black) indicates clamp connection in dikaryotic hypha.

A set of five *F. velutipes* strains (NJ6-3, NJ6-21, L11, Fv25-4, and Fv01-10) with compatible PR loci, different HD-a but similar HD-b subloci were crossed (Fig. 1). In each crossing, 0.8 cm diameter inoculum discs of PDA with actively growing mycelium (border of colony) were obtained from two compatible monokaryotic strains and placed at a distance of three cm apart from each other onto PDA plates, followed by incubation at 24 °C for 7 days. Similarly, compatibility of HD-b subloci was determined by crossing of six *F. velutipes* strains (L11, W23, Fv10-4, FV12-2, Fv-27-1, Fv-24) having different HD-b, but the same HD-a subloci (Fig. 2). Microscopic observations were done to examine the resulting colonies for the presence of clamp connections for confirmation of successful mating. For fruiting, dikaryotic mycelia were cultivated at 23 °C for 30 days in sterile plastic bags containing 52.5% cottonseed hulls, 25% wheat bran, 15% sawdust, 5% corn flour, 2% gypsum, and 0.5% ground limestone, with relative humidity of 61%. Induction of fruiting body formation to confirm the compatibility of crosses was performed at 16 °C with 90% relative humidity (Wang et al., 2016).

### Design of primers for *F. velutipes* HD subloci

Primer sets for HD subloci were designed on stretches of conserved residues within and flanking the sequences of the HD-a and HD-b subloci. Conserved nucleotides were identified based on alignments of *Hd* genes and flanking regions with ClustalW (http://www.mbio.ncsu.edu/BioEdit/bioedit.html). The efficiency of each primer set was tested by PCR in 25 μL premix (Ex-Taq kit, Takara, China), with 35 cycles and an initial melting temperature of 94 °C for 5 min followed by 94 °C 1 min; 58/63 °C (according to TM of primer set used) 40 s; 72 °C (time according to length of fragment @1,000 bp/min). PCR products were sequenced at Sangon (Shanghai, China) to confirm amplification of the correct DNA fragments.

## Results

### Identification of HD-a and HD-b subloci in *F. velutipes*

*Flammulina velutipes* strains Fv25-1, Fv25-3, Fv25-4, and Fv27-3 had been indicated to contain a HD-b sublocus but no *FvHd-a2-1* gene, leaving the existence and composition of additional HD subloci in these strains undetermined (Wang et al., 2016). Monokaryons Fv25-1 and Fv25-3 that had been obtained from the same dikaryon (protoclones of dikaryotic stain Fv25) were selected for further analysis. In order to explore more about the HD-a sublocus, a collection of mating compatible monokaryons (protoclone pairs from different dikaryotic strains) was also screened by PCR to confirm the presence or absence of the conserved *FvHd-a2-1* gene.

**Figure 3:**
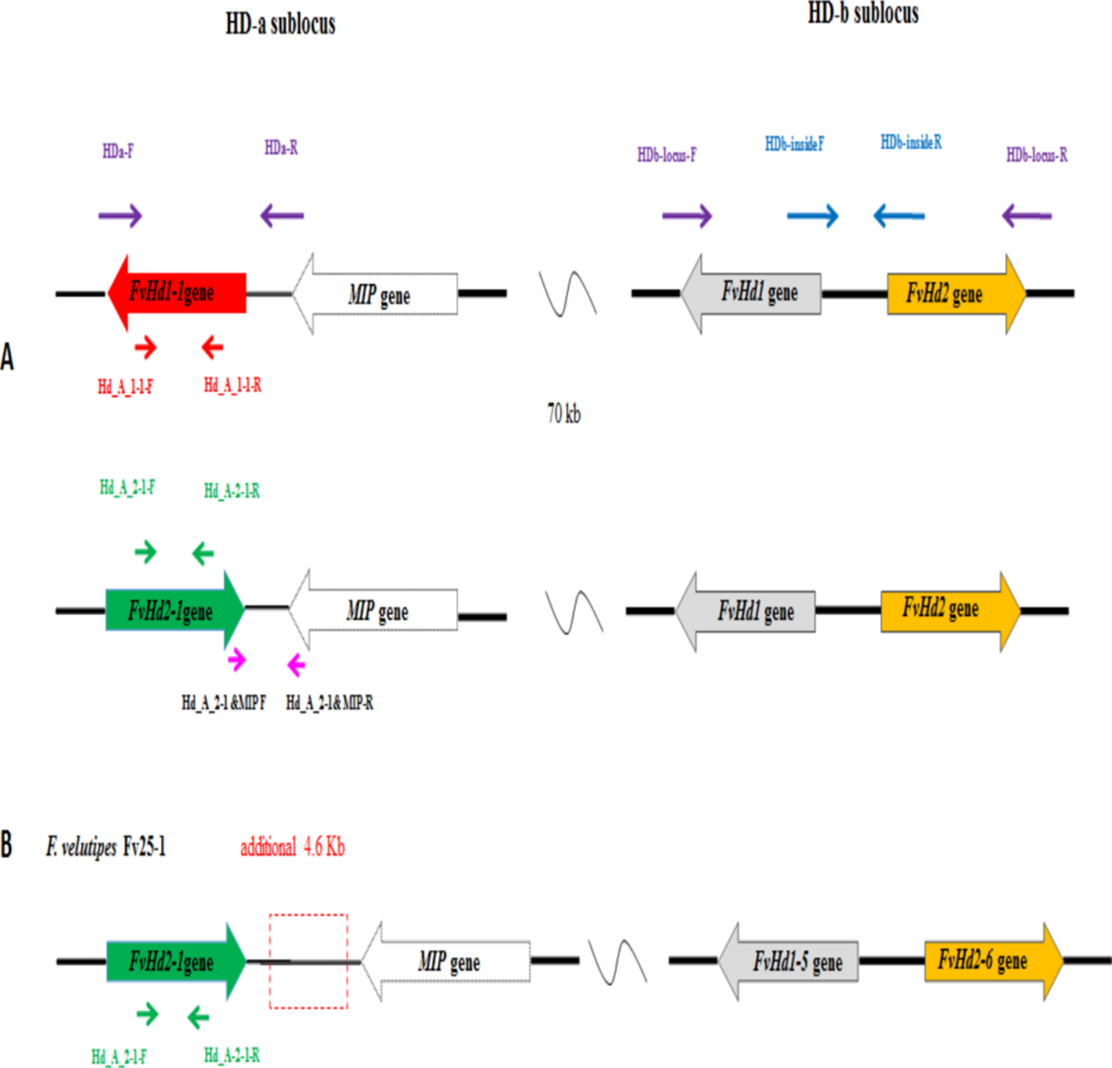
(A) Overview of the HD-a and HD-b subloci of *Flammulina velutipes*, and the locations of general (purple, blue) and gene specific (red or green) primer binding sites, (B) HD-a and HD-b sublocus of Fv25-1, with additional 4.9 kb sequence indicated in break line box.

**Figure 4:**
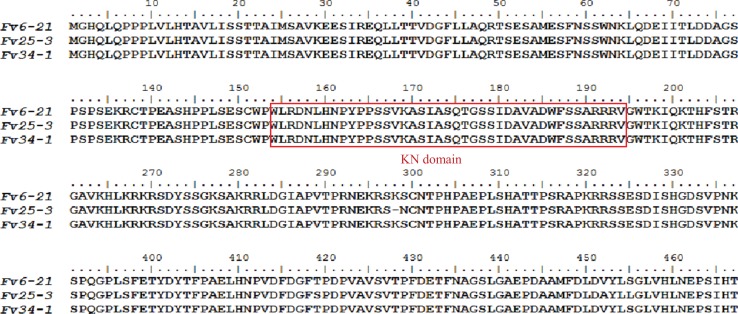
Alignment of protein sequences of FvHd_A_1-1 of *Flammulina velutipes* strains (Fv6-21, Fv25-3, and Fv34-1). Residues 154–193 form the homeobox transcription factor KN domain (a DNA-binding domain) that is marked with a red box.

Monokaryon pairs with and without a *FvHd-a2-1* gene were also obtained from dikaryon NJ6 (monokaryons NJ6-3 and NJ6-21) and Fv34 (monokaryon 34-1 and Fv34-18). Monokaryons NJ6-3 and Fv34-18 contained the *FvHd-a2-1* gene while monokaryons NJ6-21 and Fv34-1 did not. gDNA of Fv25-1, Fv25-3, Fv34-1, Fv34-18, NJ6-3, and NJ6-21 was isolated and sent for whole genome sequencing. Cleaned, 90 bp paired-end reads (six GB, or ∼170× coverage/genome) were de novo assembled to generate six draft genomes. Scaffolds that contained HD subloci were selected from the respective draft genomes based on tBLASTn and BLASTn searches with known FvHD proteins, and the *MIP* gene (van Peer et al., 2011; Wang et al., 2016; Table 1). For all six monokaryons, the HD-b and an additional HD sublocus were identified (nine scaffolds in total, Table 2). Corresponding *FvHd* and *MIP* genes were annotated by using previously identified *FvHd* and *MIP* gene and their respective protein sequences (see also Table 1). In contrast to previous PCR based analyses, the draft genome of strain Fv25-1 revealed the presence of an HD-a sublocus with the conserved *FvHd-a2-1* gene (Node-50, Table 2). Compared to other HD-a subloci with a *FvHd-a2-1* gene, the HD-a sublocus of Fv25-1 differed in sequence through a 4,588 bp insert of noncoding and repeat containing DNA between the *FvHd-a2-1* and the *MIP* gene (Fig. 3). Most probably this ∼4.6 kb insert prevented efficient amplification of *FvHd-a2-1* using primers described in Wang et al. (2016), resulting in false negative test results for presence of a HD-a sublocus in this strain. No other strains with a 4.6 kb insert at the HD-a sublocus were found. Specific primers (Table 3) were designed for the region between *MIP* and *FvHd-a2-1* or the *FvHd-a1-1* gene. Amplification and sequencing of these regions between *MIP* and the *Hd* genes (*FvHd-a2-1* or the *FvHd-a1-1*) showed that strain Fv25-1 indeed contained an extra 4,900 bp region (Fig. S5).The HD-a subloci of strain NJ6-3 and Fv34-18 were confirmed to contain the conserved *FvHd-a2-1* gene in accordance with the PCR based screens, and were found to be flanked by a *MIP* gene at similar distances (respectively, 203 or 205 bp). Strains Fv25-3, NJ6-21, and Fv34-1 also contained an HD-a sublocus, yet without the conserved *FvHd-a2-1* gene. Instead, all three strains contained the same, newly identified *FvHd1* gene; *FvHd-a1-1*. The *FvHd-a1-1* gene did not match with known *FvHd1* gene models. This gene contained four predicted introns of which two were alternatives for intron 1. Protein predictions using the two alternate intron 1 varieties resulted in different proteins with varying amino acid sequences (Fig. S1). To obtain a correct gene model and predicted protein, total RNA was extracted from strain NJ6, converted to cDNA, amplified with primers HD-a FW and HD-a RV (Table 3), cloned and sequenced. The sequenced *FvHd-a1-1* cDNA confirmed the presence of 3 introns and 4 exons, encoding a 510 amino acid protein (Fig. S1). The *FvHd-a1-1* gene and its predicted protein, like those of *FvHd-a2-1* on HD-a in other strains, was highly conserved between strains NJ6-21, Fv34-1, and Fv25-3 (Fig. 4). The *FvHd-a1-1* was located at ∼130 bp distance from the *MIP* gene, in an opposite orientation of *FvHd-a2-1* (Fig. 3). The novel *FvHd-a1-1* gene was uploaded in GenBank with accession number KU852595.1 (Table 1). Blast and alignment results showed that protein FvHD_A_1-1 contained a homeobox transcription factor KN domain (a DNA-binding domain), that is, conserved from fungi to human and plants (Fig. 4). No new genes were identified for the HD-b subloci of the eight sequenced strains, and each contained *FvHd1/FvHd2* gene pairs in the same combinations and orientations as reported before in strains W23, L11, and Fv10-4 (Table 1 in Wang et al., 2016).

**Table 3:** Primers for HD-a and HD-b subloci in different strains of *F. velutipes*. (A) Primers used to amplify genes at HD-a, (B) Primers used to amplify the whole or parts of HD-b subloci, (C) Primers used for cloning and re-sequencing of the *FvHd-a1-1* gene from cDNA.

Primer specification	Sequence (5′–3′)	Product length (bp)
**A**		
*HD-a-locus-F	CATGCCGACCATCGAATCACTTG	∼2,430
*HD-a-locus-R	CGGATATGGCGCAACGTACTATTC	
Hd-A-1-1gene-F	ACAGGAGCAATTCCATCCAGTC	∼930
Hd-A-1-1gene-R	TCACACTGCTGTCCTCATATCC	
Hd-A-2-1gene-F	GGCCACCATTATGCCAGACTTAC	∼653
Hd-A-2-1gene-R	TCCGGAAAGCGAGGTTGGAATAG	
FW-b/w HD-a 2-1&MIP	GAAGATGACGACGTGCATCG	∼250
RV-b/w HD-a 2-1&MIP	ATCCGGCCTTCATTGAGTGC	
FW-b/w HD-a1-1&MIP	CATTGTTCGCCTAAAAGAGAC	∼150
RV-b/w HD-a1-1&MIP	CTCCATTAAGTGCTGATCTAG	
**B**		
*HD-b-locus-F	CTTCTTGTCGTTCTACGTAGTAC	∼4,900
*HD-b-locus-R	AACAGGAATCGAACCTATGG	
HD-b-inside-F-ApaI	GGGCCCGGAGAGCGTGTTTTCG	∼1,063–1,090
(i) HD-b-inside-R-AflII: CTTAAGGATGCGGCGGCTAT (ii) HD-b-inside-R-BamHI: GGATCCGATGCGGCGGCTAT		
**C**		
FvHd-A-1-1cDNA-R	TTACATCCCTAAGGACGCAAAA	∼1,530
FvHd-A-1-1cDNA-F	AATGGGCCACCAATTACAGC	

**Notes:**

Any one of these Reverse primers can be used with HD-b-inside-F-ApaI for PCR HD-b conserved region between Hd-b genes.

Primers indicated with (*) are general primers for HD loci.

Nuclear localization signal, Di, and HD analyses showed that FvHD_A_1-1 contains three predicted NLS however, no Di was found in this protein (Fig. S2). FvHD_A_1-1 is clearly different from the FvHd1 on the HD-b subloci (Fig. S2).

### Functional analysis of the HD-a sublocus in mating

Interestingly, monokaryotic strains NJ6-3 and NJ6-21 contained the same set of *FvHd* genes (*FvHd-b1-5/FvHd-b2-5*) at their respective HD-b subloci (Table 2). Strains NJ6-3 and NJ6-21 have been obtained from dikaryon NJ6 that shows normal clamps and fruiting body development, and the monokaryons display normal HD mating compatibility in crosses with other strains.

**Figure 5:**
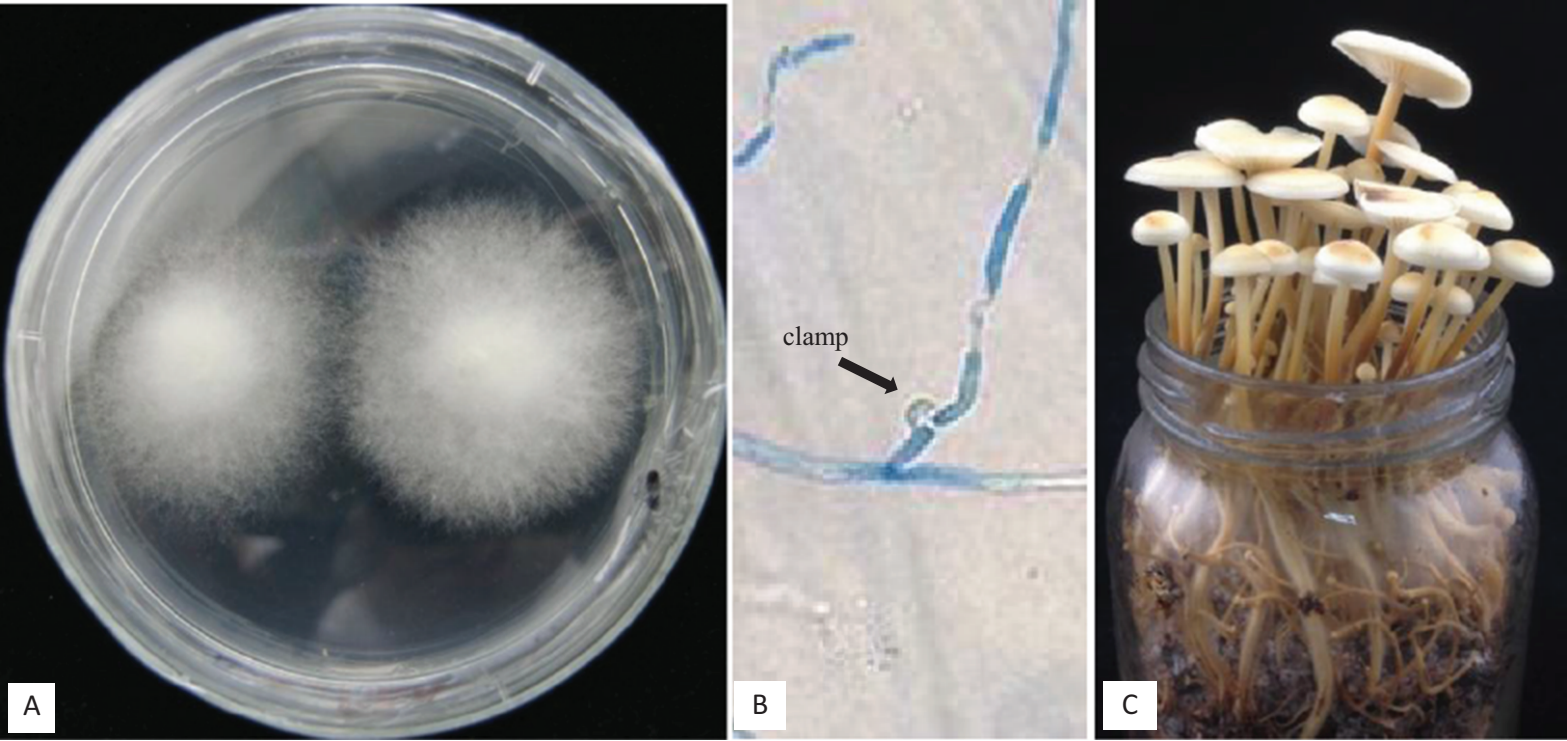
(A) Cross of Fv01 (Co1) × NJ6-3, (B) clamps formed after compatible mating of Fv01 (Co1) × NJ6-3, (C) fruiting bodies formed by a dikaryon of Fv01 (Co1) × NJ6-3, under standard conditions.

In other crosses (NJ6-3 × Fv01-10) and (L11 × Fv25-4) between monokaryotic strains containing identical HD-b subloci but different HD-a subloci resulting dikaryons produced regular clamp connections and fruiting bodies (Table 4; Figs. 1 and 5). It is likely that the conserved *FvHd-a2-1* gene at the HD-a sublocus is therefore compatible with the conserved *FvHd-a1-1* gene. Crosses between strains with identical HD-a subloci and different HD-b subloci (HD-a ═; HD-b ≠) clamp connections were formed that demonstrated the activity of HD-b sublocus in mating in accordance with previous studies (Fig. 2).

**Table 4:** Suggested renaming system for the HD mating type genes of *F. velutipes*.

Strains	Source	Monokaryon ID	Mating type	New gene names			Old gene names		
				HD-a	HD-b		Hd-a	HD-b	
L11	Fujian Edible Fungi Germplasm Resource Collection center China	L11	A1	FvHd-a2-1	FvHd-b1-1	FvHd-b2-1	FvHd2-1	FvHd1-3	FvHd2-4
W23	Fujian Edible Fungi Germplasm Resource Collection Center China	W23	A2	FvHd-a2-1	FvHd-b1-2	FvHd-b2-2	FvHd2-1	FvHd1-2	FvHd2-3
L22	Fujian Edible Fungi Germplasm Resource Collection center China	L22	A2	FvHd-a2-1	FvHd-b1-2	FvHd-b2-2	FvHd2-1	FvHd1-2	FvHd2-3
34-18	Fujian Edible Fungi Germplasm Resource Collection center China	34-18	A2	FvHd-a2-1	FvHd-b1-2	FvHd-b2-2	FvHd2-1	FvHd1-2	FvHd2-3
34-1	Fujian Edible Fungi Germplasm Resource Collection Center China	FV34-1	A5	FvHd-a1-1	FvHd-b1-5	FvHd-b2-5	FvHd_A_1-7	FvHd1-4	FvHd2-5
Fv01-1	Vanchen Mushrooms- Zhang Zhou, Fujian, China (Japanese strain)	Fv01-1	A5	FvHd-a1-1	FvHd-b1-5	FvHd-b2-5	FvHd_A_1-7	FvHd1-4	FvHD2-5
Fv01-3	Vanchen Mushrooms- Zhang Zhou, Fujian, China (Japanese strain)	Fv01-3	A5	FvHd-a1-1	FvHd-b1-5	FvHd-b2-5	FvHd_A_1-7	FvHd1-4	FvHd2-5
Fv01-11	Vanchen Mushrooms- Zhang Zhou, Fujian, China (Japanese strain)	Fv01-11	A5	FvHd-a1-1	FvHd-b1-5	FvHd-b2-5	FvHd_A_1-7	FvHd1-4	FvHd2-5
Fv01-10(Co1)	Vanchen Mushrooms- Zhang Zhou, Fujian, China (Japanese strain)	Fv01(Co1)	A5	FvHd-a1-1	FvHd-b1-5	FvHd-b2-5	FvHd_A_1-7	FvHd1-4	FvHd2-5
Fv10-4	Fujian Edible Fungi Germplasm Resource Collection center China	Fv10-4	A5	FvHd-a2-1	FvHd-b1-5	FvHd-b2-5	FvHd2-1	FvHd1-4	FvHd2-5
27-1	Fujian Edible Fungi Germplasm Resource Collection center China	27-1	A5	FvHd-a2-1	FvHd-b1-5	FvHd-b2-5	FvHd2-1	FvHd1-4	FvHd2-5
KACC42780	Mushroom Research Division, National Institute of Horticultural and Herbal Science, Rural Development Administration, Suwon, Republic of Korea	KACC42780	A3	FvHd-a2-1	FvHd-b1-3	FvHd-b2-3	FvHd2-1	FvHd1-1	FvHd2-2
Nongjin-6 (dikaryon)	Fujian Edible Fungi Germplasm Resource Collection Center China	NJ6-3	A8	FvHd-a2-1	FvHd-b1-5	FvHd-b2-5	FvHd2-1	FvHd1-4	FvHd2-5
		NJ6-21	A5	FvHd-a1-1	FvHd-b1-5	FvHd-b2-5	FvHd_A_1-7	FvHd1-4	FvHd2-5
Fv0025 (dikaryon)	Sanming Mycological Institute of Fujian Province, China	Fv25-1	A6	FvHd-a2-1	FvHd-b1-6	FvHd-b2-6	FvHd2-1	FvHd1-5	FvHd2-6
		Fv25-3	A7	FvHd-a1-1	FvHd-b1-1	FvHd-b2-1	FvHd_A_1-7	FvHd1-3	FvHd2-4

**Note:**

HD mating types were assigned to the *F. velutipes* strains in agreement with van Peer et al. (2011) and Wang et al. (2016). FvHd-a1-1 = FvHd_A_1-7 in Wang et al. (2016).

**Figure 6:**
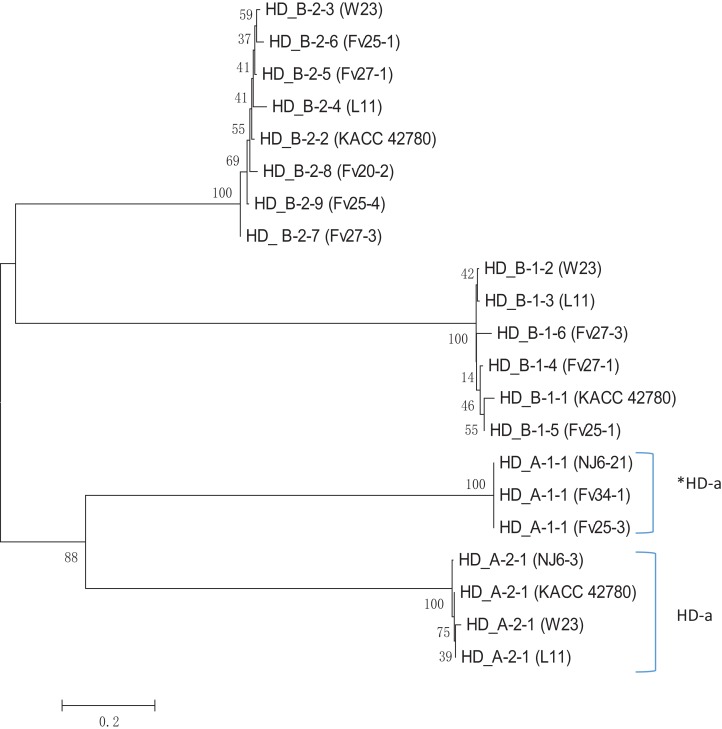
Phylogenetic tree of HD1 and HD2 protein sequences from *F. velutipes*, is demonstrating sublocus specific grouping. Proteins from HD-a subloci are indicated on the right. The HD2 protein of strain KACC 42780 is indicated that belongs to the HD-a sublocus.

Apparently, HD-a locus contains at least two “alleles,” one with a single unpaired *FvHd2* gene, and one with a single unpaired *FvHd1* gene. Relative grouping of the HD1 and HD2 proteins of the HD-a and HD-b subloci revealed two distinct clades, one containing the HD1 and HD2 proteins of the HD-a sublocus, and one containing the HD1 and HD2 proteins of the HD-b sublocus (Fig. 6). Clustering of proteins HD_A_1-1 and HD_A_2-1 in a separate clade from the HD-b sublocus, rather than clustering of all HD1 and HD2 proteins, further indicates the presence of two sets of *FvHd1* and *FvHd2* genes, belonging to two separate subloci.

### Design of primers for the HD subloci of *F. velutipes*

To determine the HD mating type of a given strain of *F. velutipes*, the two distant subloci, HD-a and HD-b, will need to be identified. To provide a quick identification method, three specific primers sets were tested for HD-a, and two sets for HD-b (Table 3). Primer set “HD-a-locus-F/HD-a-locus-R” amplified the whole HD-a sublocus including a part of the *MIP* gene, and can be used for cloning of new HD-a subloci (Fig. S4) except for strains with a HD-a sublocus similar to Fv25-1. The two other HD-a primer sets were designed on conserved sequences within the *FvHd-a1-1* and *FvHd-a2-1* gene, respectively, to easily distinguish the different HD-a subloci (Fig. 4; Fig. S3). Similarly, two sets of primers were designed on the HD-b sublocus, one for amplification of the entire HD-b sublocus, and one on highly conserved sequences within the two genes at this sublocus (amplifying the dissimilar, mating type specific parts of the *FvHd_B_1* genes and the *FvHd_B_2* genes). Amplicons can easily be sequenced in a single run and allow identification of the two different *FvHd* genes on HD-b (Fig. S4), while amplicons covering the entire HD-b can be used for cloning of the complete subloci. To evaluate the efficiency of the different primer sets in random *F. velutipes* strains, five more monokaryons (L22, Fv01-1, Fv01-3, Fv01-10(Co1), and Fv01-11) and six dikaryons (Fv18, Fv0077 Fv50599, Fv25, FV26, and Fv29) were tested in addition to the strains on which they had been designed (Table 4). All five primer sets showed reproducible results for the different *F. velutipes* strains. Either *FvHd-a2-1* or *FvHd-a1-1* was amplified for the HD-a sublocus, also even in case for the case of Fv25-1 with the large insert between *MIP* and *FvHd-a-2-1*. Complete HD-a subloci were obtained for all strains but Fv25-1 (Fig. S4). For HD-b, both primer sets generated the expected products for all tested strains. Sequencing results from amplicons of the HD-a sublocus showed that Fv01-1, Fv01-3, Fv01-10(Co1), and Fv01-11 strains contained either the *FvHd-a 2-1* or the *FvHd-a1-1* gene, together with the *MIP* gene.

## Discussion

The new data in this study provides compelling evidence for the existence of two separate, active HD mating type subloci in *F. velutipes*. HD proteins from HD-a and HD-b subloci distinguished phylogenetic sets, and cross complementation between HD proteins from HD-a and HD-b is absent. Crossing of strains with identical HD-b subloci and different HD-a subloci indicates that the two genes *FvHd-a1-1* and *FvHd-a2-1* and their respective protein products are complementary, as this invariably results in normal and fertile dikaryons. HD-a, is therefore a real HD sublocus and not a pseudo sublocus. At this moment we can not exclude the existence of additional HD subloci or further allelic variations on HD-a. However, the current number of *F. velutipes* strains from which HD loci has been identified, strongly supporting that there is no additional HD sublocus other then HD-a and HD-b.

The newly developed primer sets for the HD-a and HD-b subloci enable subcloning of these subloci from all tested *F. velutipes* strains, except for Fv25-1, for which the *FvHd-a2-1* gene can only be cloned with gene specific primers, and not whole HD-a region. While assembly errors in the draft genome of Fv25-1 can’t be fully ignored that detected a 4,588 bp insert in the HD-a sublocus corresponds with the fact that the complete HD-a sublocus region could not be amplified with the standard PCR that works on all other strains, but the separate *FvHd-a2-1* gene can be detected without problems. Also, a PCR with primers for the *FvHd-a2-1* gene flanking region generates a ∼5 kb fragment (Fig. S5). As no other deviating HD-a subloci were detected, the HD-a sublocus of Fv25-1 is likely to be an exception, and therefore the primer set for amplification of the whole HD-a sublocus can be expected to function normally in most *F. velutipes* strains.

In order to maintain clarity concerning the growing number of identified *FvHd* genes and mating types, we propose renaming of these genes and their corresponding HD loci in a more logical fashion, based on the information that at least two HD subloci exist in *F. velutipes*. During initial research (van Peer et al., 2011), it was unclear how many functional HD subloci would exist, and which and how many genes would be located on such subloci. All *FvHd1* and *FvHd2* genes were numbered chronologically following their identification, a trend that was continued by Wang et al. (2016). This has resulted in confusing name combinations for the HD (sub) loci. *FvHd1* genes at HD-b are numbered differently from their paired *FvHd2* gene and differ from the designated HD mating type. For example, strain L11 (A1B1) contains HD-b genes *FvHd1-3*/*FvHd2-4* and HD-a gene *FvHd2-1*. Moreover, *FvHd* gene numbering continues over both HD subloci, whereas the *FvHD1* and *FvHD2* genes at these subloci belong to different groups. We propose the following new guidelines to avoid further confusion: (1) The *FvHd* genes at the HD-a and HD-b subloci will be numbered independently, (2) they will be distinguished from the other sublocus with a prefix “a” or “b,” for example, *FvHd-****a****1-1* or *FvHD-****b****1-1*, (3) the HD-b sublocus contains the highest allelic variety and most often determines the HD mating type and we therefore suggest to synchronize the HD-b gene numbering with the mating type number of a strain. For example, the *FvHd* genes for strain L11 (A1B1) will be renamed to HD-b: *FvHd-b1-1*/*FvHd-b2-1* and HD-a: *FvHd-a2-1*. In case of new combinations of HD-a and HD-b genes (Table 4, compare strain L11 and Fv25-3), a new mating type is created, as the different combination can have a different functional interaction with other mating types. A complete renaming for all currently know HD loci is given in Table 4. We would encourage following this adapted naming system for any new mating type loci that are identified in *F. velutipes*.

## Conclusion

In summary, we identified the contents of HD-a and HD-b subloci in different strains and revealed that strains with similar HD-b subloci could still form normal dikaryons if they possess a different HD-a sublocus. The HD-a, is at least a di-allelic sublocus and active in mating. Based on gene arrangements and information the HD subloci, different primers sets were developed that can specifically amplify HD-a or HD-b subloci in the majority of *F. velutipes* strains. These primers sets are useful for quick identification of unknown HD mating types and mating type compatibility in *F. velutipes* strains.

## Supplemental Information

10.7717/peerj.6292/supp-1 Supplemental Information 1 A) *FvHd_a_1-1* gene with alternative but incorrect intron (blue line) and intron1 (black line) as confirmed by sequencing of cDNA, B) FvHd_A_1-1 protein sequences of the correct (510 AA), and alternative but incorrect (*) prediction.

10.7717/peerj.6292/supp-2 Supplemental Information 2 Alignment of the HD1 protein sequences from the HD-a and HD-b subloci of different F. velutipes strains. HD proteins that were present in multiple strains are included in the alignment only once. HD specific domains are indicated; dimerization motifs.

10.7717/peerj.6292/supp-3 Supplemental Information 3 (A) PCR results showing amplification of *FvHd_a_1-1* gene in different F. velutipes strains. Marker is indicated as L (red font); (B) amplification of *FvHd_a_2-1* gene in different F. velutipes strains.

10.7717/peerj.6292/supp-4 Supplemental Information 4 (A) PCR amplification of complete HD-a loci of different *F. velutipes* strains; (B) Nested PCR amplification inside the HD-b sublocus (a region in-between HD-b genes) in different *F. velutipes* strains.

10.7717/peerj.6292/supp-5 Supplemental Information 5 (A) PCR amplification of a region between *MIP* and *Hd_a_1_1*; (B) result of amplification between MIP and *Hd_a_2_1* gene in different F. velutipes strains; (C) amplification of extra region between *MIP* and *Hd_a_2-1* in 25-1 str.

## Additional Information and Declarations

### Data Availability

The following information was supplied regarding data availability: Data are available at figshare: MUKHTAR, IRUM; WANG, WEI; CHOU, TIANSHENG; JIANG, SIYUAN; LIU, XINRUI; PEER, AREND F. VAN; et al. (2018): *Flamulina velutipes* seven strains Genomes. figshare. Fileset. DOI 10.6084/m9.figshare.7531010.v1.
