# Supplementary material for: A second HD mating type sublocus of Flammulina velutipes is at least di-allelic and active: new primers for identification of HD-a and HD-b subloci
Source: PeerJ. 2019 Feb 22;7:e6292. doi: 10.7717/peerj.6292 (PMC6388666; doi:10.7717/peerj.6292)
Supplement: Supplemental Information 1 [file peerj-07-6292-s001.docx]

**
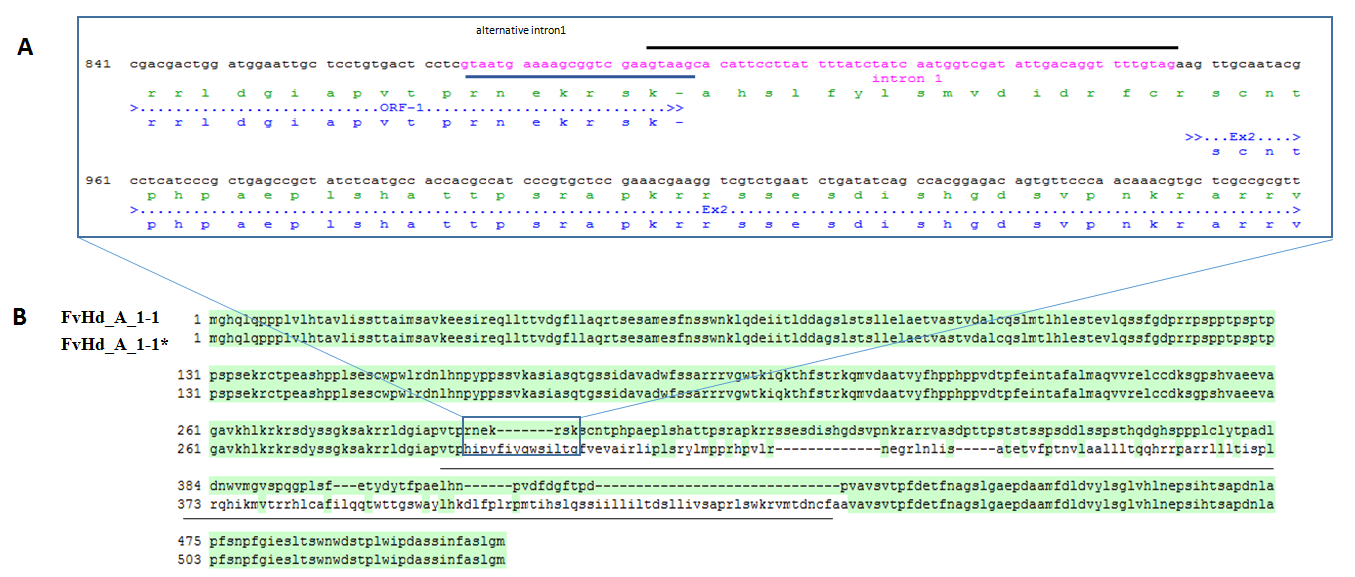
**

**Supplementary Figure 1.** (**A**) *FvHd_a_1-1* gene with alternative but incorrect intron (blue line) and intron1 (black line) as confirmed by sequencing of cDNA, (**B**) FvHd_A_1-1 protein sequences of the correct (510 AA), and alternative but incorrect (*) prediction.
