# Supplementary material for: A second HD mating type sublocus of Flammulina velutipes is at least di-allelic and active: new primers for identification of HD-a and HD-b subloci
Source: PeerJ. 2019 Feb 22;7:e6292. doi: 10.7717/peerj.6292 (PMC6388666; doi:10.7717/peerj.6292)
Supplement: Supplemental Information 2 [file peerj-07-6292-s002.docx]

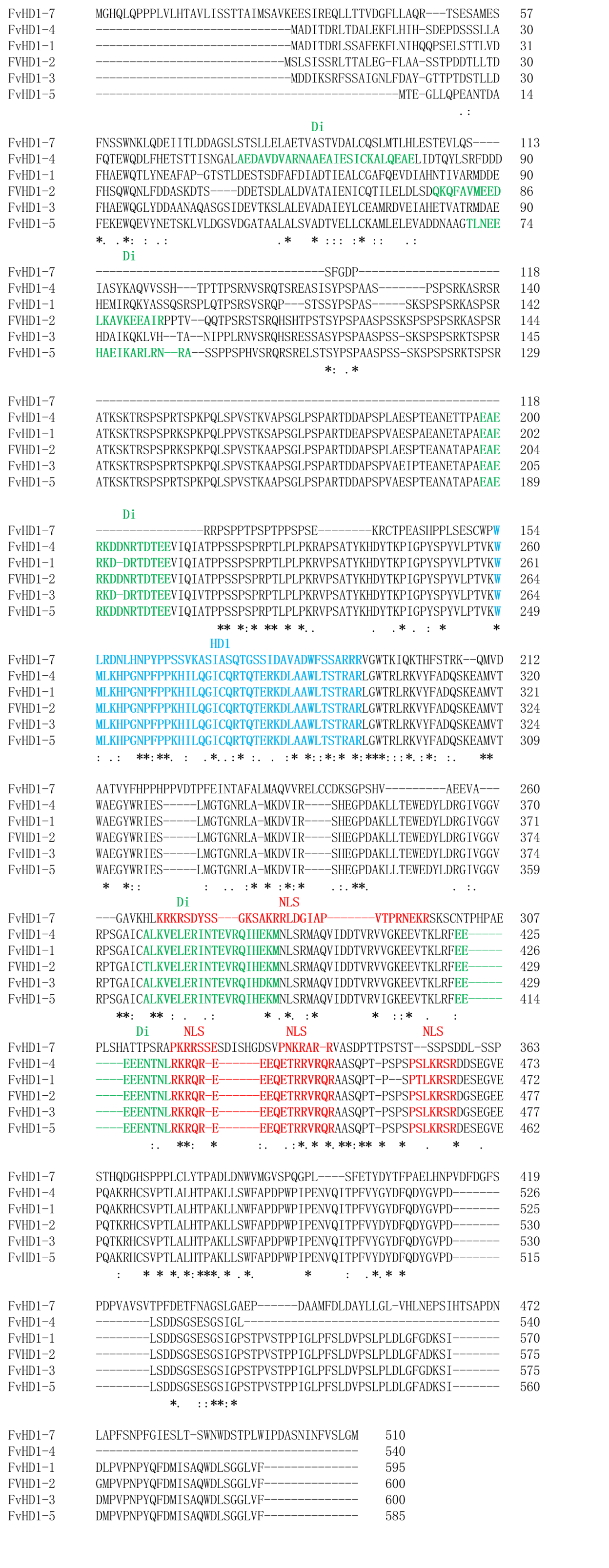


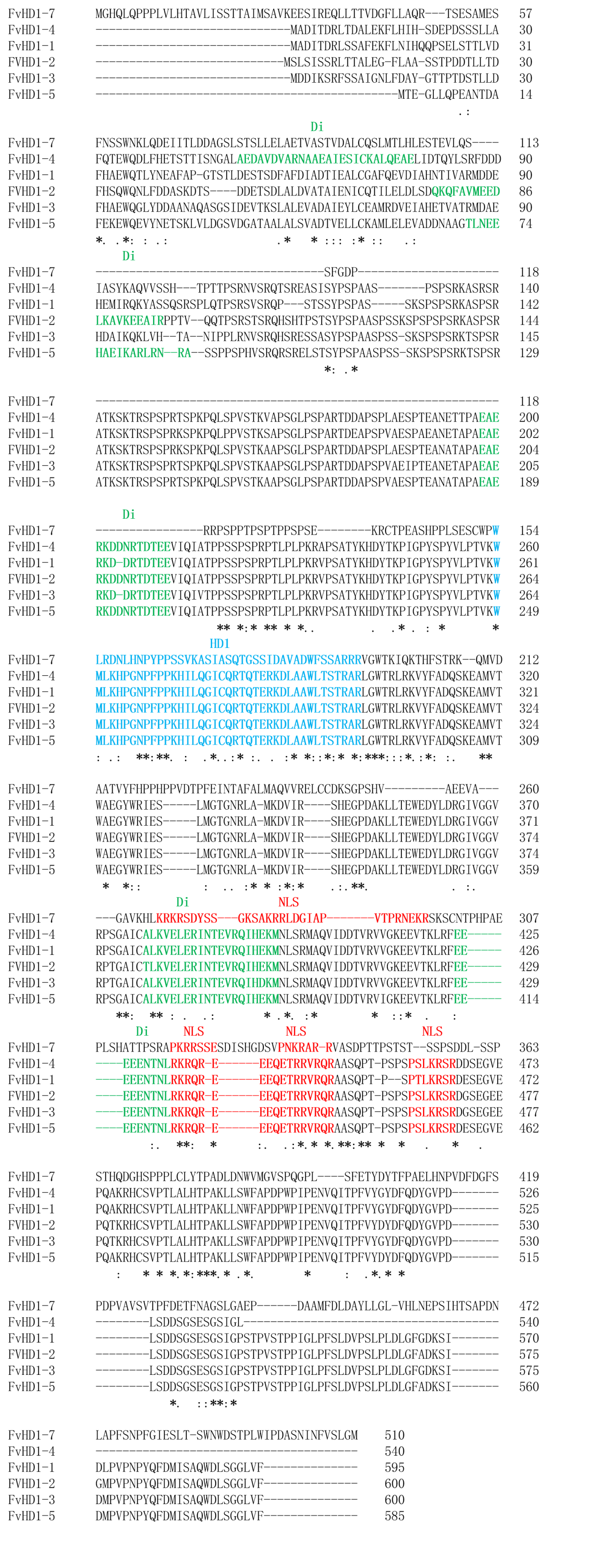


**Supplementary Figure 2.** Alignment of the HD1 protein sequences from the HD-a and HD-b subloci of different *F. velutipes* strains. HD proteins that were present in multiple strains are included in the alignment only once. HD specific domains are indicated; dimerization motifs (Di, green), homeodomains (HD, blue), nuclear localization signals (NLS, red). Conserved amino acids are marked with (*). HD1-6 is not included because of it was incomplete.
