# Supplementary material for: A second HD mating type sublocus of Flammulina velutipes is at least di-allelic and active: new primers for identification of HD-a and HD-b subloci
Source: PeerJ. 2019 Feb 22;7:e6292. doi: 10.7717/peerj.6292 (PMC6388666; doi:10.7717/peerj.6292)
Supplement: Supplemental Information 3 [file peerj-07-6292-s003.docx]

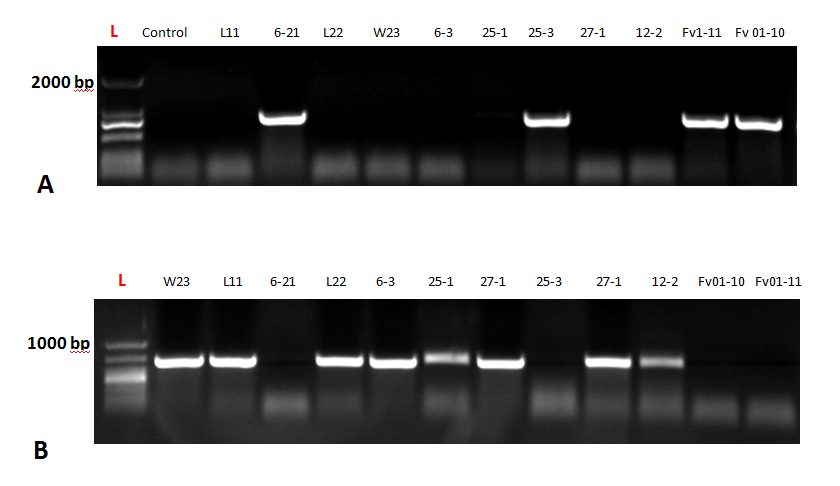


(**A**) PCR results showing amplification of *FvHd_a_1-1* gene in different *F. velutipes* strains. Marker is indicated as L (red font); (**B**) amplification of *FvHd_a_2-1* gene in different *F. velutipes* strains*.*
