# Supplementary material for: A second HD mating type sublocus of Flammulina velutipes is at least di-allelic and active: new primers for identification of HD-a and HD-b subloci
Source: PeerJ. 2019 Feb 22;7:e6292. doi: 10.7717/peerj.6292 (PMC6388666; doi:10.7717/peerj.6292)
Supplement: Supplemental Information 4 [file peerj-07-6292-s004.docx]

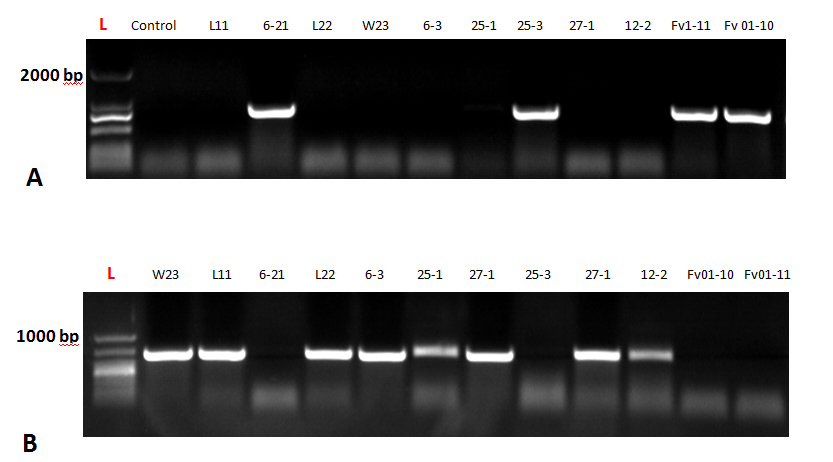


**Supplementary Figure 4**. (**A**)PCR amplification of complete HD-a loci of different *F. velutipes* strains; (**B**) Nested PCR amplification inside the HD-b sublocus (a region in-between HD-b genes) in different *F. velutipes* strains
