# Supplementary material for: A second HD mating type sublocus of Flammulina velutipes is at least di-allelic and active: new primers for identification of HD-a and HD-b subloci
Source: PeerJ. 2019 Feb 22;7:e6292. doi: 10.7717/peerj.6292 (PMC6388666; doi:10.7717/peerj.6292)
Supplement: Supplemental Information 5 [file peerj-07-6292-s005.docx]

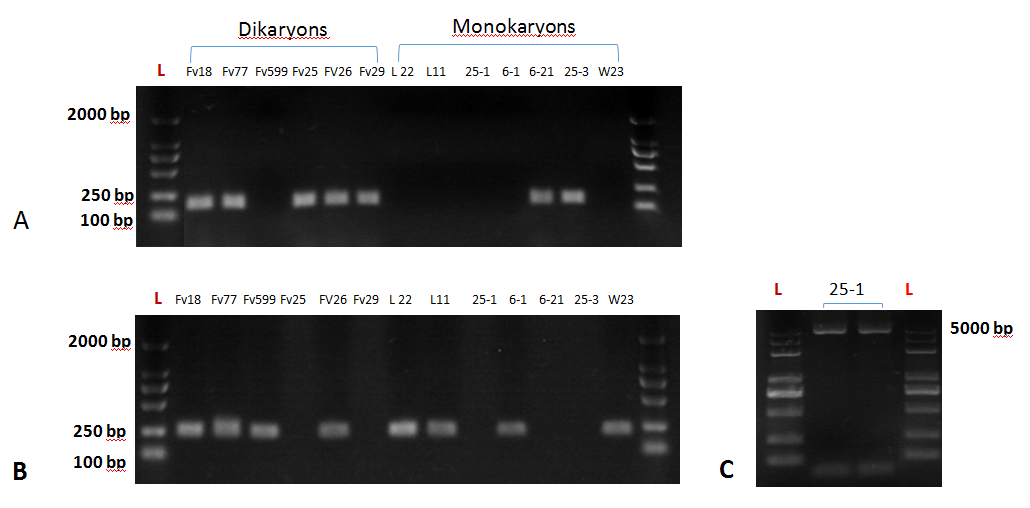


**Supplementary Figure 5**. (**A**) PCR amplification of a region between *MIP* and *Hd_a_1-1* ; (**B**) result of amplification between *MIP* and *Hd_A_2_1* gene in different *F. velutipes* strains; (**C** ) amplification of extra region between *MIP* and *Hd_a_2-1 in 25-1* strains of *F. velutipes* .
